# Maternal alcohol exposure and atopic dermatitis in offspring: A scoping review protocol

**DOI:** 10.1371/journal.pone.0354140

**Published:** 2026-07-21

**Authors:** Priyanka Renita D’Souza, Princia Rolita D’Souza

**Affiliations:** 1 Department of Psychiatry, Kasturba Medical College Mangalore, Manipal Academy of Higher Education, Manipal, India; 2 Department of Pediatric Nursing, Rohilkhand College of Nursing, Bareilly, Uttar Pradesh, India; Mansheyet El Bakry General Hospital, EGYPT

## Abstract

Maternal alcohol use is a modifiable, yet significant public health concern, causing fetal alcohol syndrome and neurodevelopmental disorders. Research has predominantly focused on the neurodevelopmental consequences of maternal alcohol consumption, but growing evidence suggests that these effects may extend to the developing immune system, potentially increasing the risk of allergic and inflammatory conditions such as atopic dermatitis in offspring, which remains underexplored. This scoping review aims to map the available literature on the association between maternal alcohol exposure (preconception and pregnancy) and atopic dermatitis in offspring, including exposure patterns, outcome measures, and hypothesized biological mechanisms. The protocol follows Joanna Briggs Institute methodology with PEO framework (Population: offspring, Exposure: maternal alcohol use, Outcome: atopic dermatitis), and has been registered prospectively on the Open Science Framework (DOI: 10.17605/OSF.IO/EWGPB). A comprehensive search of electronic databases (PubMed, Scopus, Embase, CINAHL, Web of Science) and grey literature sources will be conducted from inception through the final search date. Two independent reviewers will screen titles, abstracts and full texts, with disagreements resolved by consensus or third-reviewer arbitration. Peer-reviewed original research articles in English reporting maternal alcohol use and diagnosed atopic dermatitis in offspring will be included. Data extraction will comprise study characteristics, exposure timing and measurement, outcome diagnostic criteria and age of assessment, key findings, and reported biological mechanisms. The preliminary findings will be validated through consultation with clinical stakeholders, including experts in allergy medicine, paediatricians, dermatologists, psychiatrists and obstetricians, to maximise the review’s relevance and to identify practical implications for antenatal care and paediatric dermatology. Results will be presented through narrative synthesis, following PRISMA-ScR guidelines, to identify evidence gaps and inform future research and policy.

## Introduction

Alcohol consumption during pregnancy has adverse effects on infants and children, in addition to poor health-related outcomes in mothers [1]. Alcohol readily passes through the placenta and harms fetal development [2]. Fetal Alcohol Syndrome (FAS) refers to a severe outcome of antenatal exposure to alcohol leading to central nervous system (CNS) abnormalities, growth retardation and facial defects [3]. However, the effects of maternal alcohol consumption go beyond the defined features and can potentially have other deleterious effects on offspring. The extent of these effects may vary with the dose, frequency and duration of alcohol exposure. The genetic factors, maternal nutrition and lifestyle factors may also influence the outcomes. However, it is challenging to establish an exact dose-response relationship between the amount of maternal alcohol consumption and the severity or risk threshold caused by alcohol in the offspring [4]. The role of maternal alcohol consumption in dermatological disorders has often been overlooked and not extensively studied. Prior studies suggest that maternal alcohol exposure has a potential role in the development of atopic dermatitis in the offspring [5–7].

Atopic dermatitis(AD) is a chronic inflammatory skin condition characterised by erythematous, pruritic and scaly skin lesions localised more often to the flexural surfaces of the body [8]. It is among the most common skin disorders seen in infants and children. Atopic dermatitis develops in 45% of children within the first 6 months of life, 60% by one year of age, and 85% by the age of five [9]. It forms a part of the allergic triad along with asthma and allergic rhinitis. The clinical manifestations of atopic dermatitis precede allergic rhinitis and asthma, thus serving as an “entry point” for subsequent allergic disorders [10]. The diagnostic criteria by the United Kingdom Working Party include the presence of itchy skin with three or more of the following: asthma, rash before the age of two, flexural dermatitis, and a history of generalised dry skin. But the above criteria do not apply to infants. The extensor surfaces of the extremities, excluding the scalp, face, neck, and diaper area, are affected in infants [11]. Based on age and distribution of lesions, the phases of atopic dermatitis are categorised as infantile phase, childhood phase, and adult phase. As a chronic disorder that necessitates frequent attention, AD in children leads to distress, embarrassment, and poor self-esteem, thus leading to a lack of social relationships and poor quality of life [10].

To fully understand the association between maternal alcohol exposure and the risk of AD in offspring, it is crucial to consider the potential biological and etiopathogenetic mechanisms.

i Alcohol-induced immune dysfunction

Alcohol use induces immune dysfunction, making individuals, particularly heavy drinkers, more susceptible to various infections. The primary mechanism involves the selective inhibition of T-cell function and impairment of cell-mediated immunity (CMI), occurring even at moderate doses. Studies have demonstrated that alcohol intake can alter immunoregulatory processes, including the enhanced expression of Major Histocompatibility Complex (MHC) Class I antigens on lymphocytes [12,13].

ii T-helper cell Th1/Th2 polarisation and IgE production

The pathogenesis of AD is closely linked to an imbalanced T-helper cell response, typically involving a shift toward Th2 lymphocyte dominance in acute disease. Experimental and clinical studies suggest that alcohol exposure may exacerbate this imbalance by impairing the Th1 lymphocyte-regulated CMI response while simultaneously increasing Th2 cytokine responses like IL-4 and IL-13. This Th2-dominant environment is critical because these cytokines drive B cells to synthesise Immunoglobulin E (IgE) [7,12,14]. Epidemiological studies support this mechanistic link, reporting a positive dose-response relationship between higher maternal alcohol intake during pregnancy and elevated levels of total IgE in the cord blood of newborns [15]. This suggests a potential IgE-mediated pathway for alcohol’s influence on AD development, a distinction that warrants investigation in future research.

iii Role of ethanol metabolites and histamine release

Beyond cytokine shifts, ethanol metabolites may directly contribute to atopic symptoms. Acetaldehyde, the primary metabolite of ethanol, has been shown to induce the release of histamine from mast cells. In experimental models, alcohol consumption during pregnancy has been shown to temporarily increase histamine levels in the blood of the fetus and newborn, suggesting a transient effect linked directly to the exposure period [16].

iv Epigenetic modifications and preconception alcohol exposure

Beyond exposure during pregnancy, alcohol consumption in the preconception period may also influence offspring immune development through epigenetic mechanisms. Alcohol is a known epigenetic modifier that alters DNA methylation and histone acetylation patterns, and these modifications can be transmitted through the germline, affecting oocyte quality and early embryonic development before implantation. Disruption of methylation patterns during oogenesis may alter the programming of immune-related genes in the developing embryo, potentially predisposing offspring to Th2-skewed immune responses and atopic conditions such as atopic dermatitis [17]. Although direct human evidence linking preconception alcohol exposure to atopic outcomes remains limited, experimental models have demonstrated that periconceptional alcohol exposure can induce persistent epigenetic alterations in immune regulatory pathways [18].

These proposed pathways provide strong biological plausibility for the observed epidemiological association. However, much of the evidence relies on animal models. Therefore, this scoping review will map the extent to which these biological pathways have been investigated and reported in human studies of maternal alcohol exposure and offspring AD, identifying critical gaps.

### Rationale

The effects of maternal alcohol use on offspring represent a substantial yet modifiable public health concern. The potential association between maternal alcohol use, a modifiable risk factor and atopic dermatitis, a common childhood disorder, necessitates thorough investigation and highlights a critical opportunity for preconception and antenatal interventions. A prior systematic review by Overgaard et al. [19] suggested a preliminary association reporting a small but statistically significant positive association between maternal alcohol use during pregnancy and the development of AD in offspring. However, this review was limited by a small number of observational studies and mainly relied on binary exposure definitions (drinker vs. abstainer) without accounting for potential dose-response relationships or key exposure dimensions such as preconception alcohol use, trimester-specific exposure, and dose-frequency patterns of maternal alcohol use. Since then, the evidence has remained limited, with recent studies reporting inconsistent findings. For instance, Manjunath and Silverberg [20] reported no significant association between maternal alcohol use during pregnancy and childhood AD, while earlier cohort studies have suggested a potential positive association. In addition, variability in diagnostic criteria and broader methodological differences further limit comparability across studies. Alongside these inconsistencies, the current literature remains too diverse for a traditional systematic review aimed at establishing a definitive cause-and-effect relationship. Therefore, a scoping review methodology is the most appropriate approach to comprehensively examine exposure patterns, outcome characteristics, and hypothesised mechanisms in human studies. The primary aim of this review is to map the scope, range, and key characteristics of the existing research on the association between maternal alcohol exposure (including preconception and pregnancy) and atopic dermatitis in offspring.

## Methodology

The protocol of the scoping review, which aims to study the effects of maternal alcohol exposure on the risk of developing atopic dermatitis in the offspring, has been prospectively registered on Open Science Framework (DOI: 10.17605/OSF.IO/EWGPB). Before finalising the protocol, a preliminary search of review registries PROSPERO and OSF was conducted to identify completed or ongoing reviews with a similar population, exposure, and outcome focus. No completed or ongoing review with the same population, exposure, and outcome focus was identified at the time of the preliminary registry search. Results will be presented in accordance with Preferred Reporting Items for Systematic Reviews and Meta-Analyses extension for Scoping Reviews (PRISMA-ScR) checklist [21]. The methodological approach is adapted from The Joanna Briggs Institute (JBI) guidance [22] for scoping reviews based on the work by Arksey and O’Malley [23]. The five stages incorporated in this approach are as follows:

### A. Identifying the research question

Objectives:

- To assess the association between maternal alcohol exposure (timing, dose, pattern) and atopic dermatitis in the offspring
- To identify and map the hypothesized biological mechanisms underlying the association between maternal alcohol exposure and atopic dermatitis in the offspring
- To map the scope and characteristics of the current literature on maternal alcohol exposure and atopic dermatitis in the offspring to inform future research and policy

The review question was developed after discussion with the research team and a comprehensive assessment of existing literature on maternal alcohol use and risk of atopic dermatitis in offspring. The following research questions were formulated.

a. Is there an association between maternal alcohol exposure and atopic dermatitis in the offspring?
b. Does frequency and quantity of maternal alcohol consumption modify the risk of atopic dermatitis in offspring?
c. What biological mechanisms have been reported or hypothesised to link maternal alcohol exposure to the development of atopic dermatitis in offspring?

This scoping review will use the PEO framework to conceptualise the research questions and objectives as described in Table 1.

**Table 1:** PEO framework.

Population (P)	Offspring/Children
Exposure (E)	Maternal alcohol exposure
Outcome (O)	Atopic dermatitis

### B. Identification of relevant studies

The search strategy was initially developed for the PubMed database using the PEO framework and a combination of controlled vocabulary and free-text terms. Following peer review and validation, the strategy was adapted for each database and reviewed for accuracy, syntax, and reproducibility. Validation will include checking whether known relevant studies are retrieved by the final search strategy and documenting all final database-specific search strings in the appendix. Searches will be conducted in PubMed, Embase via Ovid, Scopus, CINAHL via EBSCOhost, and Web of Science Core Collection from database inception to the final search date. Database-specific search strategies will incorporate appropriate controlled vocabulary and free-text terms according to the PEO framework, as listed in Table 1. PubMed uses MeSH terms; Embase via Ovid uses Emtree subject headings; and Scopus and Web of Science use database-specific title, abstract, keyword, and proximity syntax. The full PubMed search strategy is presented in Table 2, and the search strategies for all other databases are provided in S1 Appendix. In addition, grey literature, including clinical trial registries, dissertations and theses, and relevant public health agency reports, will be searched. Trial registries will include ClinicalTrials.gov, the WHO International Clinical Trials Registry Platform, and the Clinical Trials Registry–India. Dissertations and theses will be searched through ProQuest Dissertations and relevant institutional/open-access repositories. Records identified from grey literature sources will be imported into the reference-management software, where possible, deduplicated, and screened using the same PEO-based eligibility criteria as those used for bibliographic database records. If export is not possible, search results will be documented manually in the grey literature search log. Additionally, the reference lists of all included studies shall be hand-searched to identify any potentially eligible articles. Studies meeting the predefined inclusion criteria will be included in the review.

**Table 2:** Search strategy for PubMed database.

Search set in PubMed Advanced Search	Concept	MeSH terms	Keywords/ free-text terms	PubMed search string
#1	Exposure: maternal alcohol exposure before or during pregnancy	“Maternal Exposure”; “Pregnancy”; “Alcohol Drinking”; “Ethanol”; “Prenatal Exposure Delayed Effects”; “Foetal Alcohol Spectrum Disorders”	maternal, mother, prenatal, antenatal, pregnancy, gestation, preconception, pre-conception, periconception, before pregnancy, before conception, prior to pregnancy, prior to conception, alcohol, ethanol, alcohol drinking, alcohol use, alcohol consumption, maternal alcohol use, maternal drinking, prenatal alcohol exposure, drinking during pregnancy, drinking in pregnancy, foetal alcohol spectrum disorder, FASD	( ( (“Maternal Exposure”[Mesh] OR “Pregnancy”[Mesh] OR “Prenatal Exposure Delayed Effects”[Mesh] OR maternal[tiab] OR mother*[tiab] OR prenatal[tiab] OR antenatal[tiab] OR pregnan*[tiab] OR gestation*[tiab] OR preconception*[tiab] OR periconception*[tiab] OR “before pregnancy”[tiab] OR “before conception”[tiab] OR “prior to pregnancy”[tiab] OR “prior to conception”[tiab] ) AND (“Alcohol Drinking”[Mesh] OR “Ethanol”[Mesh] OR alcohol*[tiab] OR ethanol[tiab] OR “alcohol drinking”[tiab] OR “alcohol use”[tiab] OR “alcohol consumption”[tiab]) ) OR ( “Fetal Alcohol Spectrum Disorders”[Mesh] OR “fetal alcohol spectrum disorder”[tiab] OR “foetal alcohol spectrum disorder”[tiab] OR FASD[tiab] OR “prenatal alcohol exposure”[tiab] OR “maternal alcohol use”[tiab] OR “maternal drinking”[tiab] OR “drinking during pregnancy”[tiab] OR “drinking in pregnancy”[tiab] ) )
#2	Outcome: atopic dermatitis/ eczema	“Dermatitis, Atopic”; “Eczema”	atopic dermatitis, atopic eczema, infantile eczema, eczema	“Dermatitis, Atopic”[Mesh] OR “Eczema”[Mesh] OR “atopic dermatitis”[tiab] OR “atopic eczema”[tiab] OR “infantile eczema”[tiab] OR eczema[tiab]
#3	Population: offspring, infants, children, adolescents, foetus/newborn	“Infant”; “Infant, Newborn”; “Child”; “Adolescent”; “Foetus”	offspring, child*, infant*, paediatric*, pediatric*, adolescent*, newborn*, neonat*, foetus, fetus	“Infant”[Mesh] OR “Infant, Newborn”[Mesh] OR “Child”[Mesh] OR “Adolescent”[Mesh] OR “Foetus”[Mesh] OR offspring[tiab] OR child*[tiab] OR infant*[tiab] OR pediatric*[tiab] OR paediatric*[tiab] OR adolescent*[tiab] OR newborn*[tiab] OR neonat*[tiab] OR foetus[tiab] OR fetus[tiab]
#4	Combined search			#1 AND #2 AND #3

### C. Selection of the eligible studies

Two independent reviewers shall be involved in the search and screening of eligible articles as described in the PRISMA-ScR flow diagram (S1 Fig). Initial screening of titles and abstracts will be conducted using the PEO framework, as outlined in Table 1, to conceptualise the eligibility criteria.

#### Population: Offspring/children.

The scoping review will include all studies examining the association in offspring, regardless of their current age, provided the AD diagnosis was made during the infantile, childhood, or adolescent phases (birth up to 18 years of age).

#### Exposure: Maternal alcohol exposure.

Studies that report any maternal alcohol use during the preconception period leading up to conception, any trimester of pregnancy, or immediately before birth, assessed by self-report, registry data or medical records.

#### Outcome: Atopic dermatitis.

Studies must report on the diagnosis of atopic dermatitis, or eczema, using established diagnostic criteria, such as the UK Working Party Criteria, Hanifin and Rajka Criteria or clinical diagnosis by a dermatologist, paediatrician, or a physician, based on symptoms, histological and laboratory findings or history by parent/guardian or registry data.

Before the formal screening process, the two independent reviewers will conduct a pilot test using a random sample of approximately 50–100 titles and abstracts. This will ensure both reviewers understand and consistently apply the PEO eligibility criteria. Any discrepancies arising during this pilot phase will be resolved through discussion, and the inclusion criteria will be refined as necessary. The titles and abstracts identified from the search will be imported into the Rayyan software to facilitate blinded, independent screening and to track the selection process as per the PRISMA-ScR. Following the initial screening, the full text of potentially eligible articles will be retrieved and independently reviewed against the eligibility criteria summarised in Table 3. Disagreements during the full-text review will first be addressed through discussion and consensus between the two reviewers. A third reviewer will be included if consensus cannot be reached about the discrepancies. The reasons for the exclusion of any full-text articles will be documented and reported in the final review. The selection process will be mapped as per PRISMA-ScR [21].

**Table 3:** Eligibility criteria.

Inclusion criteria	Exclusion criteria
- Studies examining association of maternal alcohol use during preconception period and atopic dermatitis in offspring - Studies examining use of antenatal alcohol use in mothers and its association with atopic dermatitis in offspring - Original research articles - Studies from inception to final search date - Studies published in English	- Case reports, series - Review articles - Letter to editor/editorials - Animal studies

### D. Charting the data

The data extraction process will utilise a standardised, electronic form in Microsoft Excel. Before full-scale data extraction, the charting form will be applied to a pilot sample of 5 included studies to assess inter-rater reliability, thereby ensuring clarity, consistency, and completeness in capturing the required variables. Refinements to the form will be made based on this pilot testing. One author shall extract the data, and the second author shall verify it against the original article. The verification ensures a comprehensive check for accuracy and completeness of the extracted data. A draft charting table (Table 4) will be developed to record the data obtained from each study. The second reviewer confirms all data fields in the charting table. Any discrepancies identified during verification will be resolved through discussion between the two authors.

**Table 4:** Data Charting.

- Author - Year of publication - Place of study - Study aim - Study design - Sample size - Period of exposure - Trimester specific exposure - Measurement of exposure (maternal alcohol use) - Quantity, frequency, dose, duration, pattern, severity of maternal alcohol use - Method of outcome (atopic dermatitis in offspring) assessment - Diagnostic criteria used for atopic dermatitis - Age of outcome assessment or diagnosis - Hypothesized biological mechanisms - Confounding factors adjusted for - Key findings - Limitations - Additional remarks

### E. Collating, summarising and reporting the results

The overview and characteristics of the eligible studies will be tabulated and summarised, including the author, year of publication, place of study, study design, study aim, and sample size. The included studies examining the risk of AD in offspring will be grouped according to maternal use of alcohol during the preconception period and during pregnancy. A narrative summary will integrate the following study characteristics and findings.

i. Exposure and outcome measurement: Maternal alcohol use (exposure) and atopic dermatitis (outcome) will be examined in each included study. To address potential heterogeneity, the diagnostic criteria used for atopic dermatitis, age at outcome assessment, and methods used to quantify maternal alcohol exposure, including dose, duration, frequency, timing, and pattern of use, will be extracted and mapped during data charting and synthesis. This will facilitate comparison of outcome definitions and exposure measurements across studies. Dose–response mapping will focus on how the frequency and quantity of maternal alcohol consumption (e.g., light, moderate, or heavy use, or specific grams of alcohol) were measured and whether these variables were reported to modify the risk of AD in offspring.
ii. Summary of key findings: The key findings will be summarised based on whether studies reported statistically significant positive association, no association, or an inverse association between maternal alcohol exposure and AD risk in offspring. For studies assessing antenatal alcohol exposure, the timing or trimester of exposure will also be noted and summarised.
iii. Hypothesized biological mechanism: The reported biological mechanisms associated with maternal alcohol exposure and atopic dermatitis in offspring, as discussed within the included studies, will be explored.
iv. Confounding factors: Confounding factors adjusted in the included studies, such as family history of atopy, environmental exposures, maternal smoking, and other related covariates, will be summarised.

The knowledge gap in current literature on aspects such as the specific trimester of highest risk, chronic alcohol exposure risk, lack of standardised measures for alcohol consumption, including biomarker testing and lack of longitudinal studies tracking AD incidence will be reported.

The anticipated timeline for the review is as follows:

- Search Strategy & Execution: To be finalised and executed in July 2026.
- Record screening: Title, abstract, and full-text screening are expected to be completed by August 2026.
- Data extraction and charting: Expected to be completed by December 2026, along with the synthesis of results.

### F. Consultation

The sixth stage of the JBI scoping review methodology, consultation, will be incorporated to maximise the relevance and applicability of the review findings, provide additional insights, and facilitate their dissemination. A purposive sample of approximately 5–10 experts relevant to the review topic will be invited to participate. These may include specialists and researchers in allergy and immunology, psychiatrists, obstetricians, paediatricians, and dermatologists. Participants will be identified through professional networks and academic institutions. Following the preliminary synthesis of the review findings, participants will receive a summary of the results and be invited to provide feedback through structured discussions, online meetings, or written responses. Feedback will focus on the clinical relevance of the findings, interpretation of evidence gaps, and implications for future research, clinical practice, and public health. The consultation findings will be used to contextualise and refine the interpretation of the review results, thereby enhancing the review’s practical relevance.

## Discussion and Conclusion

This scoping review will provide a structured synthesis of how maternal alcohol exposure has been examined in relation to AD in offspring, extending previous work [19] that has primarily focused on overall associations. Given the significant heterogeneity in the current evidence, a scoping review methodology is the most appropriate choice. By examining variation in exposure measurement, outcome definitions, and methodological approaches, the review will offer a clearer understanding of how this relationship has been studied and where important gaps remain. The review will also map hypothesised biological mechanisms in human studies, while recognising that the most detailed mechanistic evidence is derived from experimental or animal models. The comprehensive search of multiple bibliographic databases and grey literature sources, together with the application of established JBI methodology and PRISMA-ScR reporting guidelines, will support a rigorous, transparent, and reproducible synthesis of the available evidence.

The review may also highlight key methodological challenges within the existing literature. In particular, maternal alcohol exposure is typically assessed through self-report, which may be subject to recall and social desirability bias, especially given the stigma surrounding alcohol use in pregnancy. In addition, inconsistent adjustment for confounding factors, such as family history of atopy, environmental exposures, and maternal smoking, as well as variability in AD diagnostic criteria, may limit comparability across studies. Furthermore, only studies published in English will be included due to feasibility constraints. This restriction may introduce language bias and result in the omission of relevant evidence published in other languages. Identifying these methodological limitations will help inform the design of future epidemiological research in this field.

Maternal alcohol use is highly stigmatised, which may lead women to be hesitant to disclose their alcohol use and thus result in poor utilisation of health care services [24]. Hence, awareness and psychoeducation across antenatal, natal and postnatal care are essential. To enhance the practical implications of this review, recommendations will be informed after preliminary findings are validated through a consultation stage with clinical stakeholders, including experts in allergy medicine, paediatricians, dermatologists, psychiatrists and obstetricians. The findings may contribute to more informed clinical and public health approaches, including antenatal counselling and effective strategies aimed at reducing modifiable prenatal risk factors.

Guarantor: Priyanka Renita D’Souza

## Supporting information

S1 Figure Flowchart of selection of studies. (DOCX)

S1 Appendix Search strategy for other databases. (DOCX)

S2 Appendix PRISMA-ScR checklist. (PDF)
