# Supplementary material for: Maternal alcohol exposure and atopic dermatitis in offspring: A scoping review protocol
Source: PLoS One. 2026 Jul 21;21(7):e0354140. doi: 10.1371/journal.pone.0354140 (PMC13387542; doi:10.1371/journal.pone.0354140)
Supplement: S1 Figure — (DOCX) [file pone.0354140.s001.docx]

**Identification of studies through other sources**

**Identification of studies through databases**

Records identified from other sources (n =)

- Grey literature sources (n =)
- Citation searching (n =)

Records removed before screening:

Duplicate records removed (n =)

Records removed for other reasons (n =)

Records identified from the databases (n=)

- PubMed
- Scopus
- Embase
- CINAHL
- Web of Science

**Identification**

Records screened (title and abstracts)

Records excluded

Reports not retrieved

Reports sought for retrieval

Reports sought for retrieval

Reports not retrieved

**Screening**

Reports excluded if:

- Not met inclusion criteria
- Case reports
- Case series
- Review articles
- Letter to editor
- Animal studies
- Other language

Reports excluded if:

- Not met inclusion criteria
- Case reports
- Case series
- Review articles
- Letter to editor
- Animal studies
- Other language

Reports assessed for eligibility

Reports assessed for eligibility

Studies included in review

**Included**

**S1 Figure: Flowchart of selection of studies**
