# Supplementary material for: Maternal alcohol exposure and atopic dermatitis in offspring: A scoping review protocol
Source: PLoS One. 2026 Jul 21;21(7):e0354140. doi: 10.1371/journal.pone.0354140 (PMC13387542; doi:10.1371/journal.pone.0354140)
Supplement: S1 Appendix — (DOCX) [file pone.0354140.s002.docx]

**Scopus**

TITLE-ABS-KEY(((maternal OR mother* OR prenatal OR antenatal OR pregnan* OR gestation* OR preconception OR periconception OR "before pregnancy" OR "before conception" OR "prior to pregnancy" OR "prior to conception") AND (alcohol* OR ethanol* OR "alcohol drinking" OR "alcohol use" OR "alcohol consumption" OR "maternal drinking" OR "prenatal alcohol exposure")) OR ("fetal alcohol spectrum disorder" OR "foetal alcohol spectrum disorder" OR FASD)) AND TITLE-ABS-KEY("atopic dermatitis" OR "atopic eczema" OR "infantile eczema" OR eczema OR atopy) AND TITLE-ABS-KEY(offspring OR child* OR infant* OR pediatric* OR paediatric* OR adolescent* OR newborn* OR neonat* OR fetus OR foetus)

**Embase via Ovid**

(((exp pregnancy/ OR exp prenatal exposure/ OR exp maternal exposure/) AND (exp alcohol drinking/ OR exp ethanol/)) OR ("fetal alcohol spectrum disorder".ti,ab. OR "foetal alcohol spectrum disorder".ti,ab. OR FASD.ti,ab. OR "prenatal alcohol exposure".ti,ab.) OR ((maternal OR mother* OR prenatal OR antenatal OR pregnan* OR gestation* OR preconception OR periconception OR "before pregnancy" OR "before conception" OR "prior to pregnancy" OR "prior to conception").ti,ab. AND (alcohol* OR ethanol* OR "alcohol drinking" OR "alcohol use" OR "alcohol consumption" OR "maternal drinking").ti,ab.)) AND

(exp atopic dermatitis/ OR exp eczema/ OR ("atopic dermatitis" OR "atopic eczema" OR "infantile eczema" OR eczema).ti,ab.) AND (exp infant/ OR exp newborn/ OR exp child/ OR exp adolescent/ OR exp fetus/ OR (offspring OR child* OR infant* OR pediatric* OR paediatric* OR adolescent* OR newborn* OR neonat* OR fetus OR foetus).ti,ab.)

**CINAHL via EBSCOhost**

(((MH "Maternal Exposure" OR MH "Pregnancy" OR MH "Prenatal Exposure Delayed Effects" OR maternal OR mother* OR prenatal OR antenatal OR pregnan* OR gestation* OR preconception* OR periconception* OR "before pregnancy" OR "before conception" OR "prior to pregnancy" OR "prior to conception") AND (MH "Alcohol Drinking+" OR MH "Ethanol" OR alcohol* OR ethanol* OR "alcohol drinking" OR "alcohol use" OR "alcohol consumption")) OR (MH "Fetal Alcohol Spectrum Disorders" OR "fetal alcohol spectrum disorder" OR "foetal alcohol spectrum disorder" OR FASD OR "prenatal alcohol exposure" OR "maternal alcohol use” OR "maternal drinking" OR "drinking during pregnancy" OR "drinking in pregnancy")) AND (MH "Dermatitis, Atopic+" OR MH "Eczema+" OR "atopic dermatitis" OR "atopic eczema" OR "infantile eczema" OR eczema) AND (MH "Infant+" OR MH "Infant, Newborn" OR MH "Child+" OR MH "Adolescent+" OR MH "Fetus+" OR offspring OR child* OR infant* OR pediatric* OR paediatric* OR adolescent* OR newborn* OR neonat* OR fetus OR foetus)

**Web of Science**

TS=((((maternal OR mother* OR prenatal OR antenatal OR pregnan* OR gestation* OR preconception* OR periconception* OR "before pregnancy" OR "before conception" OR "prior to pregnancy" OR "prior to conception") AND (alcohol* OR ethanol* OR "alcohol drinking" OR "alcohol use" OR "alcohol consumption" OR "maternal drinking")) OR ("fetal alcohol spectrum disorder" OR "foetal alcohol spectrum disorder" OR FASD OR "prenatal alcohol exposure" OR "maternal alcohol use" OR "drinking during pregnancy" OR "drinking in pregnancy")))

AND TS=("atopic dermatitis" OR "atopic eczema" OR "infantile eczema" OR eczema)

AND TS=(offspring OR child* OR infant* OR pediatric* OR paediatric* OR adolescent* OR newborn* OR neonat* OR fetus OR foetus)
